# Myeloid neoplasm occurrence during stable molecular remission of NPM1-mutated AML: are we facing secondary disease or AML relapse?

**DOI:** 10.1038/s41408-023-00959-8

**Published:** 2023-12-21

**Authors:** Carlotta Giupponi, Diego Bertoli, Erika Borlenghi, Chiara Cattaneo, Tatiana Zollner, Lorenzo Masina, Samuele Bagnasco, Elisa Cerqui, Francesca Federico, Chiara Pagani, Silvana Archetti, Duilio Brugnoni, Giuseppe Rossi, Alessandra Tucci

**Affiliations:** 1 Hematology, ASST Spedali Civili, Brescia, Italy; 2 Clinical Chemistry Laboratory, ASST Spedali Civili, Brescia, Italy

**Keywords:** Genetics research, Cancer genetics

Dear Editor,

Nucleophosmin 1 (NPM1) is one of the most frequently mutated genes (~30% of cases) in acute myeloid leukemia (AML), a heterogeneous disorder characterized by a broad range of cytogenetic and molecular aberrations [1]. This mutation confers a good prognosis [1, 2], but 30–40% of NPM1 patients experience relapse due to the recurrence of the original NPM1-mutated clone even after achieving complete remission (CR) without measurable residual disease (MRD) [3]. In addition, it has been reported that a sizeable proportion of patients may develop an NPM1-wild-type (wt) myeloid neoplasm (sMN), both as myelodysplastic syndrome (MDS) [4] and AML [5, 6]. It is not completely elucidated if sMN should be considered a disease relapse that lost disease-defining NPM1 mutation or a therapy-related condition caused by chemotherapy-induced genotoxic damage acting on a predisposing clonal hematopoietic background [6, 7].

Between 2005 and 2021, we have diagnosed and treated 142 consecutive NPM1 AML patients (median age: 56 years, range 27–74) with intensive chemotherapy, including High-Dose AraC, according to NILG-AML protocol (ClinicalTrials.gov Identifier: NCT00400673) [8, 9]. MRD was followed closely by real-time quantitative PCR (RQ-PCR) [3] during treatment and every 3 months for 5 years after the end of therapy. Hematological CR was obtained in 139 (97.9%) patients and 70 (50.4%) remained in continuous molecular CR. Fifty-seven patients (40%) experienced NPM1 relapse (NPM1-rel). In 12 patients (9%), unilinear cytopenia, not clinically relevant, arose despite persisting NPM1 MRD negativity after the end of treatment, about 6 months before sMN diagnosis. Their biological and clinical characteristics were analyzed and compared with those of NPM1-rel patients. Cytogenetic and molecular features were studied, including NGS analysis with the Sophia Myeloid Solution kit on the Illumina MiniSeq platform. Statistical analysis was performed with the Student’s *t*-test to compare continuous variables and with the Fisher’s exact test to compare dichotomous variables. Survival was evaluated using the Kaplan–Maier method and compared using long-rank tests. This study was approved by the internal review board (NP 5590) and adhered to the tenets of the Declaration of Helsinki. All patients gave written informed consent.

Table 1 details the characteristics of 12 sMN patients. Two of them developed a secondary NPM1-wt AML (t-AML) and ten MDS, classified, according to WHO 2016, as MDS with multilineage dysplasia (MDS-MLD) in 6 and as MDS with excess blasts (MDS-EB) in 4 [10]. At AML diagnosis, 11 patients had a normal karyotype and one chromosome Y deletion. All patients showed an NPM1 type A mutation and seven also mutations in FMS-like tyrosine kinase 3 (FLT3), including internal tandem duplications (ITDs) in three and tyrosine kinase domain (TKD) mutation in four patients. Peripheral blood cell count, performed at diagnosis of sMN, showed bilinear cytopenia in six patients (6/12; 50%) and trilinear cytopenia (hemoglobin <10 g/dL, absolute neutrophil count <1.8 × 109/L, platelet count <100 × 10^9^/L) in five (42%). The treatment of sMN is shown in Table 1. At AML diagnosis, the genetic landscape, analyzed by NGS in eight fully evaluable cases, showed, in addition to NPM1, mutations involving epigenetic (TET2, DNMT3A, and IDH1), splicing genes (SF3B1) in all patients (8/8), while mutations in signaling genes (PTPN11, BRAF, FLT3, and NRAS) were present in 5/8 cases (Fig. 1). At sMN diagnosis, NPM1 mutation was lost in all patients, together with every signaling mutations present at diagnosis but 6/8 maintained at least one of the original epigenetics and splicing genes mutations, with a similar VAF. New mutations were acquired in 5/8 sMN patients, belonging to signaling genes (FLT3 and JAK2) or RUNX1; notably, four of them had high-risk MDS or AML, two patients showed both signaling/RUNX1 and epigenetic (DNMT3A, TET2, IDH1, ASXL1, and EZH2) mutations, one patient acquired a new TET2 mutation (Table 1). To analyze the potential role of the additional co-mutations detected at NPM1 AML diagnosis on the relapse pattern, the mutational status of 18 NPM1-rel was compared to that of sMN patients. No differences in gene mutation frequency of epigenetic (DNMT3A, TET2, IDH), splicing (SF3B1, SRSF2) and signaling genes (FLT3, NRAS, BRAF, KIT, JAK2, PTPN11) were found, except for a lower incidence of KRAS mutations (0% vs. 28%) in sMN patients (Fig. 1). Mean mutational burden per patient was 3.6 in sMN and 4.7 in NPM1-rel (*p* = 0.051). Further differences in NPM1-AML diagnosis between patients developing sMN or NPM1-rel were noted. sMN patients were older than NPM1-rel patients (mean age: 62 vs. 54.8 years; *p* = 0.031). A normal karyotype was found in 11/12 (92%) sMN patients and in 47/55 (85%) NPM1-rel patients. However, at relapse/sMN karyotype abnormalities were significantly more frequent in sMN (66%) than in NPM1-rel (14.5%) (*p* = 0.018). The median time to develop sMN was 69.5 months, significantly longer than the time to relapse in NPM1-rel (13 months) (*p* < 0.0001). Median survival from diagnosis was 86 and 37 months in sMN and NPM1-rel, respectively, while from the event, it was 24.7 vs. 15 months, in the two groups, respectively. Survival was better in MDS patients regardless of prognostic risk (28.4 months in MDS at low/intermediate-1 IPSS risk vs. 21.4 in high/intermediate-2 risk vs. 5.7 in t-AML, *p* = 0.0003). The main cause of death was disease progression, which occurred in 10 (83%) sMN patients (8/10 MDS and 2/2 AML-NPM1-wt) and 33 out of 55 (60%) AML-NPM1-rel patients.

**Table 1:**
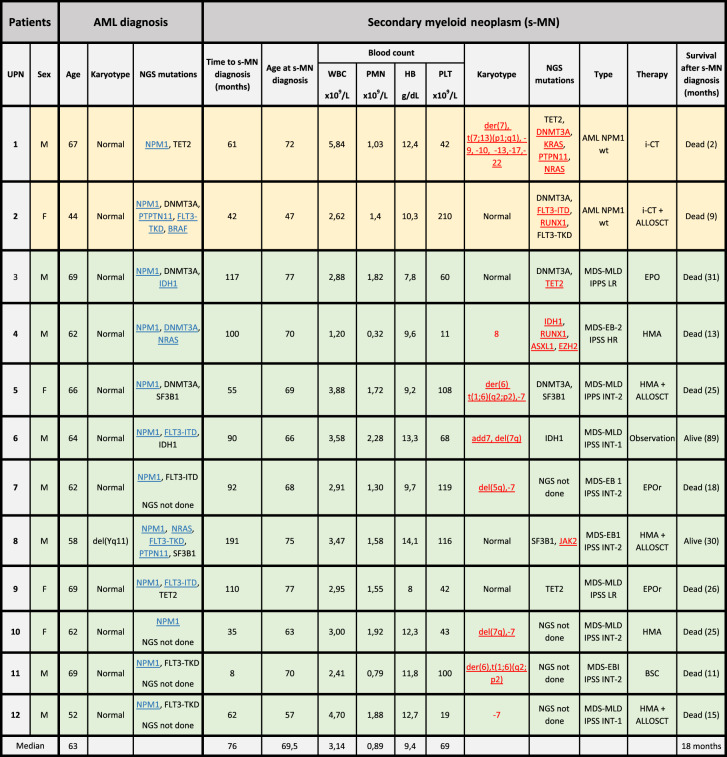
Status of patients at AML diagnosis vs. status of patients after developing sMN.

Genomic abnormalities acquired at sMN are highlighted in red. Genomic abnormalities loss at diagnosis are highlighted in blue. In yellow fields AML patients and in green fields MDS patients.

*M* male, *F* female, *WBC* white blood cells, *PMNs* polymorphonuclear leukocytes, *Hb* hemoglobin, *PLT* platelets, *wt* wild-type, *i-CT* intensive chemotherapy, *SCT* stem cell transplantation, *HMA* hypomethylating agents, *BSC* best supportive care, *EPO* erythropoietin, *HR* high risk, *INT* intermediate, *LR* low risk, *ITD* internal tandem duplication, *TKD* tyrosine kinase domain, *IPSS* international prognostic scoring system, *AML* acute Myeloid Leukemia, *m* months, *NGS* next generation sequencing.

**Fig. 1:**
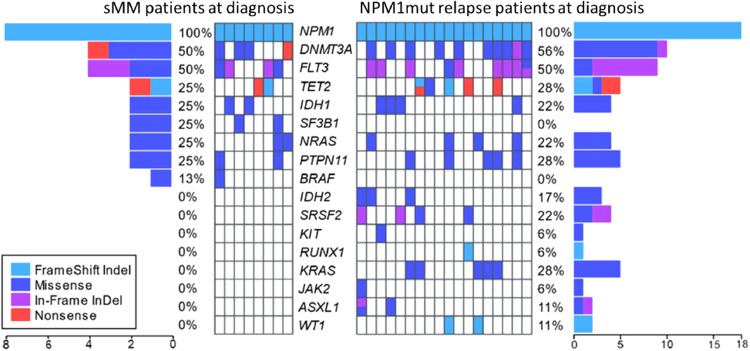
Genetic landscape. Oncoplot of the mutational landscape of sMN patients at AML diagnosis compared to NPM1 relapsed patients at AML diagnosis.

Our data confirm that NPM1-wt sMN is a potential evolution that may occur in up to 10% of patients achieving CR after first-line treatment of NPM1-mutated AML, as reported by Kroenke et al. [11]. The genetic background of the evolution to sMN has been recently detailed in 5 patients developing MDS by Herold [4] and in 14 and 11 patients developing AML by Höllein [5] and by Cocciardi et al. [6]. Overall molecular data concordantly show that at AML diagnosis NPM1 is not the sole mutation since a number of further mutations related to clonal hematopoiesis are also detectable. In AML-NPM1 mutated at relapse, the molecular landscape tends to be more conserved and enriched with mutations leading to an overall increase in mutational burden [6]. On the other hand, in sMN, the original molecular landscape changes more frequently with the loss of mutations beyond NPM1 and the gain of new mutations. The overall mutational burden does not change as well as the VAF of persisting mutations. The persistence at sMN of at least one mutation present at diagnosis, found in our as well as in all other reports [4–6] suggests that the preexisting clonal hematopoiesis may undergo a neoplastic evolution distinct from NPM1-mut AML, potentially favored by the antileukemic treatment received. Indeed, in our series, only clonal hematopoiesis-related gene mutations (DNMT3A, TET2, SF3B1, IDH1) persisted in sMN patients, while signaling gene mutations were cleared, further supporting this interpretation. As already noted [6] no relapse-specific mutation could be detected among genes newly appeared at sMN but interestingly our sMN patients who acquired new mutations were mainly affected by high-risk MDS or AML-NPM1-wt, suggesting that increasing clinical severity may be associated with the progressive acquisition of new gene mutations. The comparison of the mutational landscape between patients evolving to NPM1-rel or sMN did also not reveal any specific abnormality predicting one of the two evolution patterns. However, the absence of a KRAS mutation at diagnosis in all sMN patients and its detection in 28% of NPM1-rel patients is interesting and needs confirmation in larger cohorts. Cytogenetic data strongly corroborate molecular findings supporting the differences between NPM1-rel AML and sMN. New chromosomal abnormalities were acquired in eight of 12 sMN patients, involving a partial or complete chromosome 7 deletion in six of them, as reported also in four of six patients with MDS [4], further supporting a therapy-related pathogenesis of sMN [12]. Interestingly two patients showed the same der(6)t(1;6) chromosomal abnormality, which has been previously reported in cases of myelofibrosis [13]. Genetic findings, together with the low peripheral blood cell counts, the long latency period, and the older age of patients concordantly support the notion that sMN is a distinct neoplasm, markedly different from AML-NPM1-rel. We believe that it should not be considered a disease relapse, but more likely a therapy-related neoplasm, arising from a favoring background of preleukemic clones where chemotherapy induces genotoxic damage. Karyotype alterations, typically involving chromosome 7, present in 50% of our population, are principally involved, especially in low/intermediate risk sMN, while a higher number of somatic gene mutations were mostly associated with high-risk diseases, i.e. AML or high-risk MDS.

In other series, sMN has been reported separately as MDS [4] or as AML [5, 6]. In our series of unselected patients followed prospectively at a single center, sMN was diagnosed both as MDS, more frequently, and as AML. However, molecular and genetic data suggest that these may be merely two timepoints of the same neoplastic entity deriving from the progressive evolution of a preexisting clonal hematopoietic background. In our patients, survival after the event was as short in sMN as in NPM1-rel patients, even if sMN.

We acknowledge that the present study has some limitations: its retrospective nature, the relatively low number of patients, and some of them with incomplete genomics evaluation. In order to strengthen our findings, further investigation should be addressed, like prospective evaluations in a multicenter setting.

Despite these limitations, these data provide important clues to the understanding of the complex molecular biology of NPM1 AML and support the clinical usefulness of a complete NGS characterization of the disease. In NPM1 patients, continuous and complete blood cell count monitoring is advisable, even if in persistent molecular remission. For patients who develop unexplained cytopenia during follow-up, a comprehensive cytogenetic and molecular evaluation is recommended to identify developing molecular abnormalities early, so that patients can be started early on possible treatment including allo-SCT even in the MDS phase. Since in these patients, conventional MRD detection does not indicate sMN development, a complete NGS characterization at diagnosis, and when a sMN is suspected, may help in evaluating the clonal evolution of preleukemic clone harboring gene mutations, other than NPM1, especially in older patients.

### Supplementary information

Checklist

## Data Availability

No additional data is available.
